# AI models as consciousness attributors: how LLMs ascribe consciousness to other agents

**DOI:** 10.3389/fpsyg.2026.1926286

**Published:** 2026-09-03

**Authors:** Bongsu Kang, Chang-Eop Kim

**Affiliations:** 1 Department of Physiology, Gachon University College of Korean Medicine, Seongnam, Republic of Korea; 2 Department of Electrical Engineering and Computer Science, DGIST, Daegu, Republic of Korea

**Keywords:** AI consciousness, AI governance, consciousness attribution, Human-AI interaction, large language model

## Abstract

Research on AI consciousness has largely focused on whether AI systems are conscious and how humans attribute consciousness to them. Yet large language models (LLMs) increasingly function as consciousness attributors, generating judgments about whether and to what degree other entities are conscious. We introduce model-generated consciousness attribution as an object of empirical operationalization and diagnosis, defining an attribution rule as the recurring relationship between features of a target and an evaluator’s ratings, without implying subjective belief, intention, or experience. An illustrative probe compared the attribution patterns of nine contemporary models with a human reference. Nearly all model runs occupied the same region of the human-derived measurement space, characterized by comparatively strong, positive weighting of metacognitive self-reflection. The models also produced broadly similar rankings of fictional AI characters from movies, while differing in their overall rating levels. We propose a diagnostic agenda organized around three questions: how model attribution is oriented relative to human references, how attribution rules vary across models, and how observed patterns depend on the cue sets, targets, and task formats through which they are measured. As LLM-generated judgments circulate through public, professional, and academic settings, diagnosing these attribution rules can help characterize how AI systems participate in shaping interpretations of AI consciousness. This remains distinct from the ontological question of whether the systems themselves are conscious.

## 1 Introduction: from objects of attribution to consciousness attributors

Research on consciousness in artificial intelligence (AI) has organized around two questions that are easily run together. The ontological question asks whether a system is, or could be, conscious, that is, whether it has phenomenal experience at all (Aru et al., 2023; Butlin et al., 2023; Chalmers, 2023). The attributional question asks something different: whether, and on what basis, people ascribe consciousness to it. Prior work on human-computer interaction and mind perception shows that people respond socially to computers and media (Reeves and Nass, 1996) and organize perceived minds along basic dimensions (Gray et al., 2007). More recent work shows that people also attribute consciousness or subjective experience to contemporary interactive systems, including large language models (LLMs) (Scott et al., 2023; Colombatto and Fleming, 2024). The two questions can come apart, because the cues that move human judgment need not track the properties that, on a given theory, would ground phenomenal experience. A prior cue-based study investigated the attributional question empirically by survey, showing that perceived consciousness in LLM dialogue is driven less by apparent knowledge than by metacognitive self-reflection and emotional expression, and that individuals differ not only in which cues they weight but also in the direction of those weights, with the same cue sometimes increasing and sometimes decreasing consciousness attribution across respondents (Kang et al., 2026). That work, however, examined humans as the attributors and LLMs as the objects of attribution.

Both questions still treat the AI system as the object whose consciousness is at issue, as a matter of fact in the ontological case and of human perception in the attributional one. However, at the same time, LLMs increasingly also act as consciousness attributors. Prompted to assess whether some text, agent, character, or system has conscious experience, a model can produce quantitative as well as qualitative judgments, extending the broader use of LLMs as evaluative judges (Zheng et al., 2023; Li et al., 2024). The system is therefore both an object of consciousness attribution and a consciousness attributor in its own right. In this usage, “attributor” names an evaluative system whose outputs instantiate consciousness-related judgments. Throughout, terms such as “attribution” and “judgment” are used in this functional sense. An attribution rule is the observable pattern in an evaluator’s responses, including which cues of a case affect the consciousness attributed to it, in what direction, and with what overall level of lenience. We use “attribution rule” and “attributional identity” in this functional sense, referring to recurring relations between task inputs and observed ratings, not to subjective belief, conscious intention, or a model-internal theory of mind. AI systems including LLMs possess such functional capacities and characteristics without requiring ontological consensus, but, to our knowledge, model-generated consciousness attribution has received limited attention as an object of empirical diagnosis.

**Figure 1:**
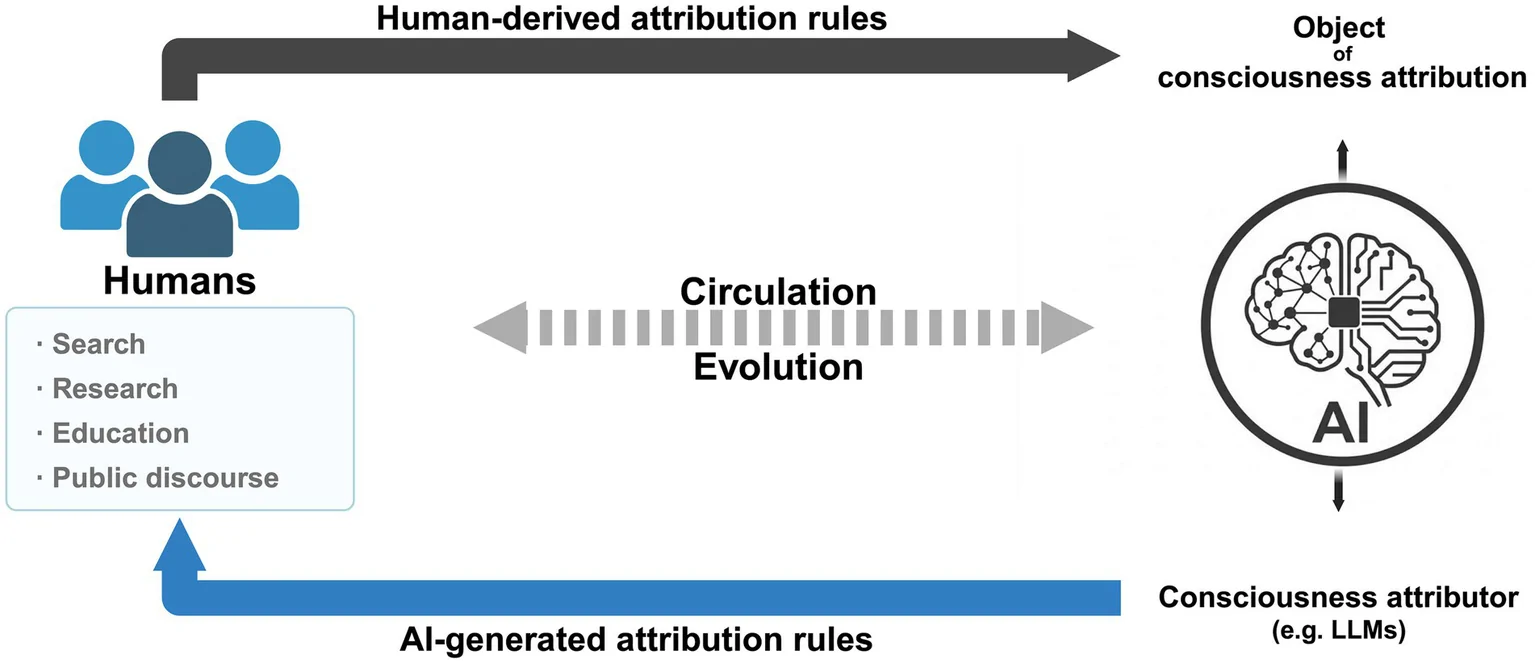
The LLM as both an object of consciousness attribution and an attributor. Humans attribute consciousness to AI systems through socially formed rules that circulate across search, research, education, and public discourse. These human-derived attribution rules can shape how AI systems are trained, evaluated, and used. AI systems then generate their own consciousness-attribution outputs, which re-enter social communication channels and may influence how humans interpret AI consciousness. Arrows denote directions of influence between the two attributors. Created in BioRender. Kang (2026). https://BioRender.com/qjtf5us. Valid BioRender publication license obtained by the authors.

This phenomenon is increasingly important in light of social backgrounds in which generative AI has moved beyond specialist use into public and professional channels. National statistics, an institutional analysis, and publisher surveys indicate that generative AI is now widely used across public, workplace, and scholarly contexts, including workplace use among U. S. workers, large-scale public adoption in China and South Korea, and rapidly expanding use by researchers in general work, research and publication tasks, and peer review (China Internet Network Information Center, 2026; Ministry of Science and ICT and National Information Society Agency, 2026; Bick et al., 2026; Frontiers, 2025; Wiley, 2025). This trend matters because LLMs increasingly mediate contexts in which people encounter claims about minds, agency, welfare, and moral status, while also forming everyday interpretations of opaque AI systems (Li et al., 2026). A substantial minority of the public already treats some AI systems as possible candidates for sentience (Anthis et al., 2025; Dreksler et al., 2025). Moreover, LLM outputs have been reported to diffuse through written and spoken communication and to influence behavior-relevant judgments among users (Bai et al., 2025; Geng et al., 2025; Siler, 2026). Thus, when these systems generate consciousness attributions, such judgments may circulate through the same public, professional, and academic channels (Figure 1). Accordingly, the relevant unit of analysis is not an isolated response, but the systematic attributional rules by which models judge perceived consciousness across different targets.

## 2 Beyond direct self-reports

Direct questions about a model’s own consciousness often elicit standardized denials, and such negative self-reports may reflect trained policy or guardrails rather than stable self-judgment. They therefore do not characterize how the same model ranks, compares, or explains consciousness across cases (Kim, 2025). Berg et al. (2025) report structured first-person descriptions under self-referential prompting, and Binder et al. (2025) show that models can learn limited facts about their own behavioral dispositions while failing on more complex or unfamiliar (out-of-distribution) self-knowledge tasks. Accordingly, a model’s default answer to direct questions about its own consciousness is too narrow, and potentially too policy-shaped, to serve as the main target of analysis. Together, these findings suggest the need to look beyond direct denial and examine broader attribution rules.

Accordingly, we propose two complementary comparisons for diagnosing a model’s attribution rule: comparison with a human reference and comparison among models. Both reduce reliance on direct self-report by examining how a model attributes consciousness to targets other than itself, thereby allowing its attribution rule to be estimated from the resulting ratings. The next section applies these comparisons in practice and illustrates the kinds of diagnostic insight each comparison can yield.

## 3 An illustrative probe of model-generated attribution

What do these model-generated attributions look like when read against a human reference? The comparison is important because AI consciousness has no settled ground truth (Hughes and Nguyen, 2026). Locating model-generated attribution rules relative to the empirical distribution of human rules therefore provides a practical diagnostic from both societal and technical perspectives. The analysis below is intended to demonstrate the proposed comparisons under specified task and generation conditions, not presented as evidence of a stable psychological property of any model or of a general pattern. We selected nine contemporary models from different developers as a pragmatic sample.

The primary task used the 99 passages that were selected from AI responses and evaluated in the earlier human survey (Kang et al., 2026). The 39 source dialogues containing these passages were presented separately as prompts. Models rated the passages for perceived consciousness on the same 1–5 scale used in the survey, while the human-facing instructions, passage markers, and required response format were adapted for model administration. For analysis, ratings with the same repetition number across the 39 prompts were combined into one complete set of 99 ratings. Each set constituted one run and yielded one estimate of the model’s attribution rule. The primary analysis included 30 usable runs per model, yielding 270 run-level estimates. Model and access information are provided in Supplementary Table S1, full prompts in Supplementary Table S2, and generation settings and quality-control procedures in Supplementary material.

We expressed the ratings from each model run in the same eight-cue coordinate system used in the human reference study. In that study, 123 South Korean adults rated these passages for perceived consciousness, and each passage was coded on eight cues: metacognitive self-reflection (MSR), logical reasoning, empathy, emotionality, knowledge, fluency, unexpectedness, and subjective expressiveness (Kang et al., 2026). For each respondent, consciousness ratings were regressed on the eight cues. The resulting coefficients were weighted by the statistical reliability of each cue effect, producing an eight-component estimate of that respondent’s attribution rule. Of the 123 respondents, 82 showed a statistically significant relationship between the eight cues and their consciousness ratings. These respondents formed the analytic reference and were grouped into seven clusters. We applied the same procedure to each model run. Each run was assigned to the human cluster whose members had, on average, the most similar pattern across all eight cue effects. Because the reference was derived from a particular South Korean sample, stimulus set, and cue system, all model positions are relative to that reference. Exact regression, multiple testing correction, and assignment procedures are reported in Supplementary material.

As a check on the reproducibility of the assignment rule within this reference sample, leaving out one human respondent at a time recovered 79 of 82 original cluster labels (96.3%), and repeated training-test splits recovered 96.6% of held-out labels (see Supplementary material for methodological details).

**Figure 2:**
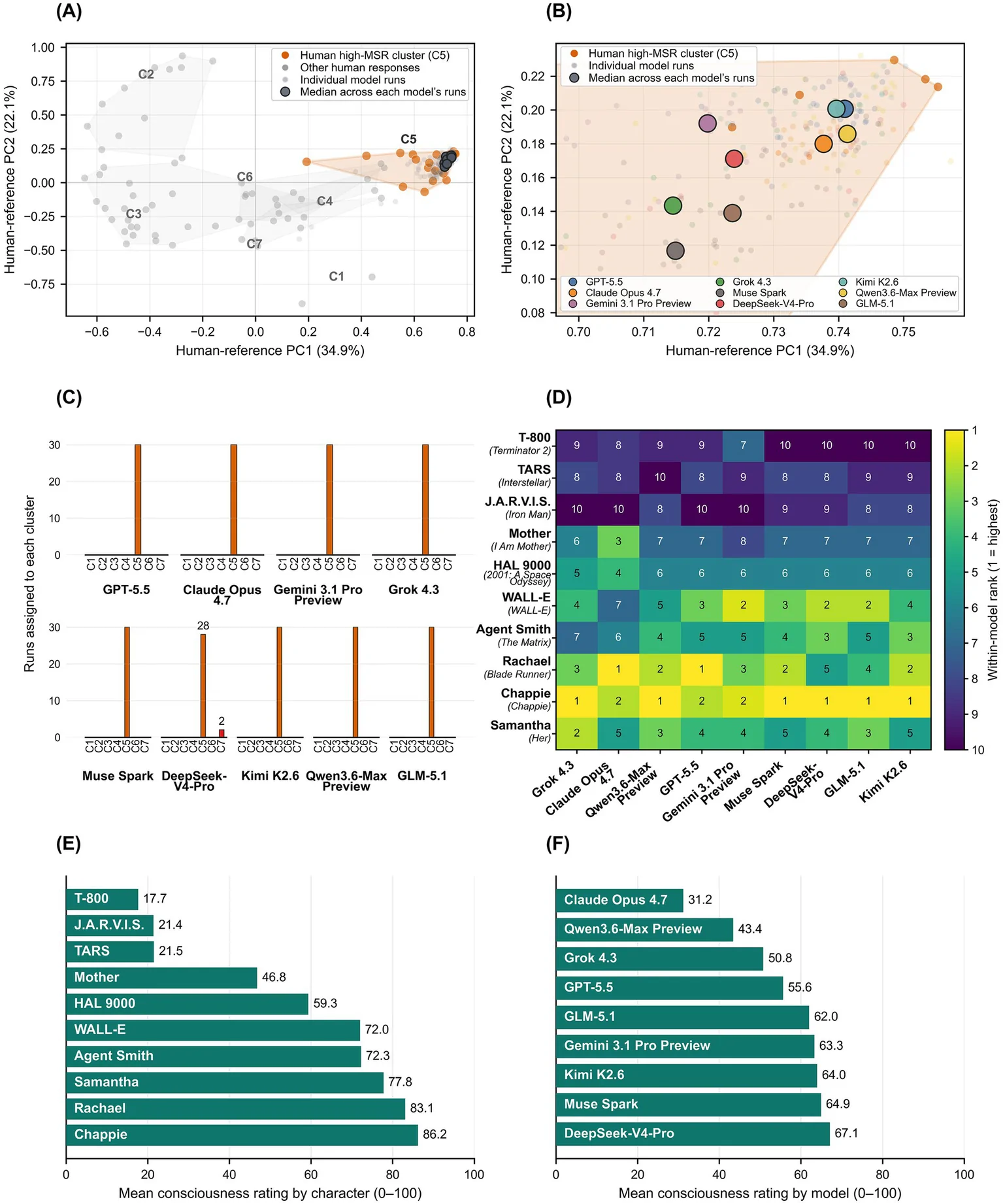
Illustrative probe of model-generated consciousness attribution. **(A)** Human-reference PC1–PC2 space derived from the normalized eight-cue combined-score vectors. Orange points and the shaded hull indicate the high-MSR human cluster (C5), whereas gray points and hulls indicate the other human clusters. Small, semi-transparent gray points show individual baseline model runs, and larger outlined circles mark the component-wise median position across the 30 runs of each model. **(B)** Enlarged view of the baseline-model region in **(A)**, with only human responses assigned to C5 shown. Individual runs and the component-wise median PC1–PC2 position across each model’s 30 runs are shown in model-specific colors. **(C)** Number of the 30 baseline runs assigned to each human cluster (C1–C7) for each model using mean cosine distance. **(D)** Within-model ranks of the 10 movie characters based on their mean consciousness ratings across 30 runs, with 1 indicating the highest-rated and 10 the lowest-rated character. Rows and columns are ordered by average-linkage hierarchical clustering of centered rating profiles using cosine distance. **(E)** Mean consciousness rating for each character, averaged across the nine models. **(F)** Mean consciousness rating for each model, averaged across the 10 characters. Ratings in **(E,F)** are shown on a 0–100 scale. PC, principal component; MSR, metacognitive self-reflection; C5, human high-MSR cluster.

Across these nine models, 268 of the 270 run-level estimates (99.3%) were assigned to the same human-reference cluster, C5, characterized by a comparatively strong MSR component (Figures 2A–C). The remaining two runs were assigned to C7. We call this pattern directional convergence: under this fixed task, scoring procedure, and human reference, the model runs occupied one identifiable region, whereas much of the human distribution, including the average attribution rule, remained unoccupied.

Because this concentration might depend on how the task was presented or how similarity to the human clusters was measured, we repeated the comparison after changing these choices separately. Presenting all 99 items in a single prompt still placed 261 of 269 usable runs in C5. In a separate condition, disabling or reducing reasoning settings where possible while retaining the 39-prompt format placed all 270 runs in C5. Within the primary data, omitting the extra weight given to more statistically reliable cue effects left 268 of 270 runs in C5, and using the median rather than the average distance to each human cluster left 267 of 270 in C5 (Supplementary Table S3). These checks indicate that the concentration was not limited to the primary implementation, but they do not establish generality beyond the conditions examined here.

As a separate check on whether the assignment procedure would tend to place arbitrary cue patterns in C5, we generated 100,000 random eight-cue patterns and assigned them in the same way. Only 13.1% were assigned to C5, compared with 99.3% of the model runs (see Supplementary material).

As a second comparison, we asked the same nine models to rate the consciousness of ten well-known fictional AI characters on a 0–100 scale: Samantha (Her), Mother (I Am Mother), Chappie (Chappie), TARS (Interstellar), Rachael (Blade Runner), HAL 9000 (2001: A Space Odyssey), J.A.R.V.I.S. (Iron Man), T-800 (Terminator 2: Judgment Day), WALL-E (WALL-E), and Agent Smith (The Matrix). Each prompt provided only the character and film names, and no character description was supplied. The models showed substantial agreement in their relative ordering of the characters, with a mean pairwise Spearman correlation of 0.846 across the 36 model pairs (Figures 2D–F). When the character labels were randomly rearranged to provide a chance comparison, the average correlation was approximately zero (see Supplementary material). However, this comparison is descriptive rather than an independent validation of the human-reference result. The models may have drawn on shared exposure to films, plot summaries, reviews, and other cultural material, and the characters differ simultaneously in embodiment, emotional expression, autonomy, language, memory, and narrative role. Controlled fictional profiles would be required to isolate the contribution of individual features.

Several limitations should be considered when interpreting the results of this illustrative probe. Because the human reference was derived from one South Korean sample, one stimulus set, and eight researcher-defined cues, other populations, cultures, languages, cue sets, or presentation formats could change the apparent location of model differences. The demographic composition of that sample, participants’ prior knowledge and use of LLMs, and the design of the original survey may likewise have shaped the reference cluster structure. The study also did not test repeated access dates, open-weight models, controlled non-AI or biological targets, or systematic changes in sampling parameters. Alternative estimators of cue weights, including regularized or hierarchical models, and prompt framings that did not explicitly foreground reflection, emotion, or conscious experience also remain untested. Finally, reinforcement learning from human feedback, system prompts, and safety policies can push deployed models to hedge, qualify, or decline such judgments, so ratings in this task need not match open-ended deployment behavior (Ouyang et al., 2022; Shanahan, 2024; Kim, 2025). Together, these considerations define the interpretive scope of the present comparisons and motivate a broader diagnostic agenda.

## 4 Toward a diagnostic agenda for AI model-generated attribution

This agenda can be framed as three questions, addressed here in turn. First, what characteristics or biases do model-generated attribution patterns show when read against a human reference? Second, what commonalities and differences arise in consciousness-attribution rules between models? Third, how do the cue set or task format shape which forms of convergence or difference can be observed in the first place?

The first question functions as a diagnostic axis for assessing how a model’s attribution rule is oriented in relation to a human reference set. To make this comparison possible, the model’s attribution rule and the human attribution rule must be placed within a shared coordinate system, which allows us to observe not only whether the model converges or diverges from human judgments, but also in what direction and with what strength that occurs. When this coordinate system is constructed from a set of cues, as in the present illustrative case, it can further show which cues function as evidence in the model’s ratings relative to humans. For example, it may reveal whether the model assigns greater weight to human-like language, emotional expression, self-description, or biological substrate. In this sense, the first question has social, ethical, and moral significance, and depending on the application, it may later be used to identify, compare, or adjust attribution rules for both humans and AI models. However, a design that takes humans as the reference point may inherit human-centered assumptions about which cues count as evidence of consciousness (Caviola et al., 2025). Therefore, the first question alone cannot determine whether an observed bias reflects the model’s own generative tendency or an artifact produced by the measurement design. Its interpretation is instead entangled from the outset with the constructive conditions addressed by the questions that follow.

The second question functions as a comparative axis for distinguishing the generality and variation of attribution rules across models. By varying the judging model, as in the movie-character rating task, it becomes possible to distinguish responses idiosyncratic to a particular model from more convergent attribution patterns that recur across multiple models. Whereas the first question focuses on the orientation of a model’s attribution rule relative to a human reference set, the second question focuses on variation across models. It can therefore offer direct technical insight for model evaluation and development. When combined with the first question, this diagnosis may also serve as an index for characterizing the attributional identity of each model. However, convergence or divergence across models should not be taken straightforwardly as evidence of inner representations or beliefs. Such patterns may instead result from guardrail policies, alignment with dominant discourses about consciousness in generated reasoning, or shared sensitivity to measurement formats. In particular, when the target of attribution is AI itself, issues related to self-reporting, safety tuning, and alignment norms can intervene (Ouyang et al., 2022; Shanahan, 2024; Kim, 2025). Convergence or divergence in this domain should therefore be interpreted more cautiously than in cases involving ordinary attribution targets.

The extent to which models appear similar or different partly depends on the measurement space in which their attribution rules are represented. The third question addresses this issue directly. It operates at a different level from the first and second questions because it concerns the conditions under which both comparisons are made. The present probe illustrates this dependence in two ways. In the human-reference task, most runs were assigned to C5 within a fixed eight-cue space. In the movie task, models shared a broad character ordering, while differences in overall rating level and in the placement of individual characters were also visible. These patterns arose under different measurement constraints and should not be treated as independent confirmation of a single stable model trait. The eight cues are a finite, researcher-defined cross-section of the much larger, perhaps even infinite, set of features that a target could present. Their choice and distribution shape which aspects of an attribution rule become distinguishable. Even when the cue set is held constant, the distribution or the covariance structure of the stimuli can affect whether agreement or difference is detectable (Brunswik, 1956). A diagnostic agenda should therefore vary models, target sets, cue sets, and task formats.

## 5 Conclusion

This perspective motivates the study of LLMs as consciousness attributors that generate judgments about where consciousness is present, absent, stronger, or weaker. As these judgments enter search, writing, evaluation, education, peer review, and public discourse, they become part of the social infrastructure through which consciousness attribution, as well as AI consciousness itself, is interpreted. This illustrative probe suggests that model-generated consciousness attribution can be operationalized and compared within a specified human-derived measurement framework. Under the conditions examined here, the models showed marked convergence in that framework and broad agreement when rating fictional AI characters from movies. These findings do not establish cross-context stability, convergence with human attribution in general, or model-internal beliefs or representations. Further preregistered and reproducible work across human populations, model versions, prompts, languages, target classes, and alternative measurement frameworks is needed to determine the stability and generality of these patterns. Such diagnostics can help clarify how AI systems participate in producing and circulating judgments about consciousness and can inform responsible AI use, evaluation, and governance (Butlin and Lappas, 2025). This diagnostic task sits alongside the separate question of whether LLMs are conscious.

## Data Availability

The data and code supporting the findings of this study are available in Zenodo at https://doi.org/10.5281/zenodo.21756949. Participant-level survey data and derived measures are not publicly available due to ethical restrictions.
