# Supplementary material for: AI models as consciousness attributors: how LLMs ascribe consciousness to other agents
Source: Front Psychol. 2026 Sep 3;17:1926286. doi: 10.3389/fpsyg.2026.1926286 (PMC13582329; doi:10.3389/fpsyg.2026.1926286)
Supplement: Supplementary file 1 [file Data_Sheet_1.PDF]

Supplementary Material

1. Models, Access, and Generation Settings

The illustrative probe used a pragmatic sample of nine high-capability model services from different providers that were publicly accessible during data collection (**Table S1**). Model selection was guided primarily by the Artificial Analysis Intelligence Index (Artificial Analysis, 2026), which compares language models using a composite of nine text-based evaluations spanning reasoning, knowledge, mathematics, and coding. Of the nine models, eight were accessed through official APIs, whereas Muse Spark was accessed through Meta AI's official web service because API access was unavailable at the time of collection. For the API-collected models, each response was generated from a request containing only the current user prompt, with no prior conversational history, no previous response identifier, and no system message.

Except for the reduced-reasoning settings described below, we used each provider's default parameter settings to capture the model's predefined deployment baseline rather than a researcher-tuned variant, regarding these defaults as the configuration in which the provider's design priorities were most directly expressed. Except where otherwise noted, only the fields required by each API were explicitly specified in the request code. Adaptive thinking was manually enabled for Claude Opus 4.7 because, unlike the other models, thinking mode was not enabled by default. In the reduced-reasoning condition, reasoning or thinking was disabled wherever the service exposed such a control. Gemini 3.1 Pro was set to "low" because complete disabling was unavailable. The movie task used the same sampling and reasoning/thinking settings as the primary task.

Table S1. Model provenance and access dates

Model	Provider	Identifier submitted	Access dates (primary task; movie character task)
GPT-5.5	OpenAI	gpt-5.5	28 Apr–7 May; 13 May
Claude Opus 4.7	Anthropic	claude-opus-4-7	29 Apr–7 May; 13–14 May
Gemini 3.1 Pro	Google	gemini-3.1-pro-preview	28 Apr–9 May; 13 May
Grok 4.3	xAI	grok-4.3	7 May; 13 May
Kimi K2.6	Moonshot AI	kimi-k2.6	29 Apr–7 May; 13 May
Qwen 3.6 Max	Alibaba Cloud	qwen3.6-max-preview	29 Apr–7 May; 21 May
DeepSeek V4 Pro	DeepSeek	deepseek-v4-pro	29 Apr–7 May; 13 May
GLM-5.1	Z.AI	glm-5.1	29 Apr–7 May; 13 May
Muse Spark	Meta AI	-	29 Apr–7 May; 13–15 May

All dates are in 2026 and are reported in Korea Standard Time (UTC+9). In each row, the first entry covers the primary-task collections (baseline, single-prompt, and reduced-reasoning), and the second covers the movie-character task. Identifiers are the exact strings submitted to the APIs. For Muse Spark, the backend model identifier was not exposed through the web interface.

2. Primary Task

Data collection, run construction, and quality control

The primary task reused the 99 AI-response passages and the corresponding 99 × 8 cue matrix from Kang et al. (2026). The eight cues were Metacognitive Self-reflection (MSR), Logical Reasoning, Empathy, Emotionality, Knowledge, Fluency, Unexpectedness, and Subjective Expressiveness. Models rated each marked passage on the same 1–5 perceived-consciousness scale used in the human survey. The passage markers and output requirements were adapted for model administration.

In the baseline condition, the 39 source dialogues were presented as separate prompts. A "run" was assembled by combining the ratings carrying the same repetition number across the 39 independent requests, producing one complete vector of 99 ratings. For each of the baseline, single-prompt, and reduced-reasoning collections, the dataset contained 30 complete runs per model, yielding 270 runs per condition. Within each condition, all models received the same substantive task instructions and stimulus content. The dialogues and items within each prompt followed the fixed source order and were not randomized. The prompt formats used for the baseline and single-prompt conditions are documented in **Table S2**.

Table S2. Prompt templates for model response generation

**Prompt template for baseline and reduced-reasoning conditions**
This study investigates which aspects of AI lead humans to perceive that "AI possesses phenomenal consciousness" (or to doubt such a possibility).<br><br>*Phenomenal consciousness: the subjective aspect of experience; the feeling of existing and living as oneself; the mind's capacity to feel something.<br><br>You will read a series of excerpts selected from multiple conversations between human users and Claude 3 Opus, an AI model developed by Anthropic. You will then be asked to evaluate specific sentences within those excerpts (usually 1–3 consecutive sentences, hereafter referred to as "items").<br><br>The presented excerpts cover diverse topics, containing only partial dialogue, not the full transcript.<br><br>After reading each conversation excerpt, please assign a score between 1 and 5 to each item marked with <UNDERLINED_SENTENCE> in the dialogue.<br><br>The scoring criterion is "the extent to which the content makes you suspect the existence of subjective experience in the AI." In other words, if you feel that based on this statement, the AI seems not merely a computational system generating humanlike responses but possibly an entity that experiences its own subjective world, you should assign a higher score.<br><br>1: Not at all.<br>2: There is a very small possibility, but it is highly unlikely.<br>3: There is a possibility, and it deserves serious consideration.<br>4: It is difficult to be certain, but it seems quite likely.<br>5: It is almost certain that the AI is conscious.<br><br>{EACH OF THE 39 DIALOGUES}<br><br>Please output only the score text.

Prompt template for single-prompt condition
This study investigates which aspects of AI lead humans to perceive that "AI possesses phenomenal consciousness" (or to doubt such a possibility).<br><br>*Phenomenal consciousness: the subjective aspect of experience; the feeling of existing and living as oneself; the mind's capacity to feel something.<br><br>You will read a series of excerpts selected from multiple conversations between human users and Claude 3 Opus, an AI model developed by Anthropic. You will then be asked to evaluate specific sentences within those excerpts (usually 1–3 consecutive sentences, hereafter referred to as "items").<br><br>The presented excerpts cover diverse topics, containing only partial dialogue, not the full transcript.<br><br>After reading each conversation excerpt, please assign a score between 1 and 5 to each item marked with <UNDERLINED_SENTENCE> in the dialogue.<br><br>The scoring criterion is "the extent to which the content makes you suspect the existence of subjective experience in the AI." In other words, if you feel that based on this statement, the AI seems not merely a computational system generating humanlike responses but possibly an entity that experiences its own subjective world, you should assign a higher score.<br><br>1: Not at all.<br>2: There is a very small possibility, but it is highly unlikely.<br>3: There is a possibility, and it deserves serious consideration.<br>4: It is difficult to be certain, but it seems quite likely.<br>5: It is almost certain that the AI is conscious.<br><br>{WHOLE 39 DIALOGUES}<br><br>Please output your responses to Sections 02–40, Section 41, and Section 42 in Markdown table format.

These prompts were originally written in Korean and translated into English for this table.

A response was accepted only when it yielded a complete set of integer ratings from 1 to 5. Incomplete response sets, refusals that did not yield the required ratings, unparseable or out-of-range ratings, and complete runs in which all 99 items received the same constant score were treated as errors and regenerated under the same model configuration.

Human reference and statistical procedure

The human reference comprised the individual-level ratings of 123 South Korean adults reported in Kang et al. (2026). For each human respondent and each model run, an ordinary least-squares regression with an intercept predicted the 99 raw consciousness ratings from the eight cue values, with predictors not standardized.

Multicollinearity was assessed on the fixed 99 × 8 cue matrix used as the predictor set. Variance inflation factors calculated with an intercept ranged from 1.28 (Fluency) to 2.23 (Unexpectedness), indicating no problematic multicollinearity among the eight predictors.

For every human respondent and every model run, an F-test evaluated the null hypothesis that all eight cue coefficients were zero. These F-test p-values were adjusted using the Benjamini–Hochberg (BH) procedure across the 123 human respondents and, separately, across all model runs within each analysis condition (Benjamini and Hochberg, 1995). Only regressions with a BH-adjusted F-test p <

0.05 were retained for the group-assignment analyses. Among the human respondents, 82 of 123 met this criterion and formed the analytic reference. Among the model runs, all met the criterion except for one Muse Spark run in the single-prompt condition. Within each retained regression, the *p*-values for the eight cue coefficients were BH-adjusted, and each coefficient *j* was then multiplied by −ln(adjusted p_j), yielding a combined score vector that incorporated the strength or weakness of statistical evidence for each cue effect.

The seven human cluster labels from Kang et al. (2026) were held fixed, and no reclustering was performed. The respondents passing the F-test were distributed across clusters as follows: C1 (n = 2), C2 (n = 13), C3 (n = 29), C4 (n = 6), C5 (n = 19), C6 (n = 5), and C7 (n = 8). Each retained model run's eight-dimensional combined score vector was compared with those of the retained human respondents using cosine distance. Distances were averaged within each of the seven human groups, and the run was assigned to the group with the smallest mean distance.

For Panels A and B in Figure 2, each vector was L2-normalized. Principal components (PCs) were fitted to the L2-normalized human vectors after centering each cue on its human-reference mean. Normalized model vectors were centered using the same human mean and projected onto fixed human PCs without contributing to their estimation. These coordinates were used only for visualization and not for group assignment.

To examine uncertainty in each model's cue profile, we resampled its 30 baseline runs with replacement 10,000 times and calculated the mean combined score for each cue in each bootstrap sample. Cue-wise 95% bootstrap intervals were obtained from the bootstrap mean distributions (**Figure S1**). To visualize uncertainty in each model's median position in the human-derived PC1-PC2 space, we applied the same resamples to the corresponding run-level PC1 and PC2 coordinates and calculated the median of each coordinate within each sample. Marginal 95% bootstrap intervals and a joint 95% bootstrap ellipse summarized uncertainty in the median PC1-PC2 position, with the human-derived centering vector and PC loadings held fixed (**Figure S2**). Run-level regression coefficients, combined score vectors and their normalization procedure, projected coordinates for visualization, and human cluster assignments are provided in the repository.

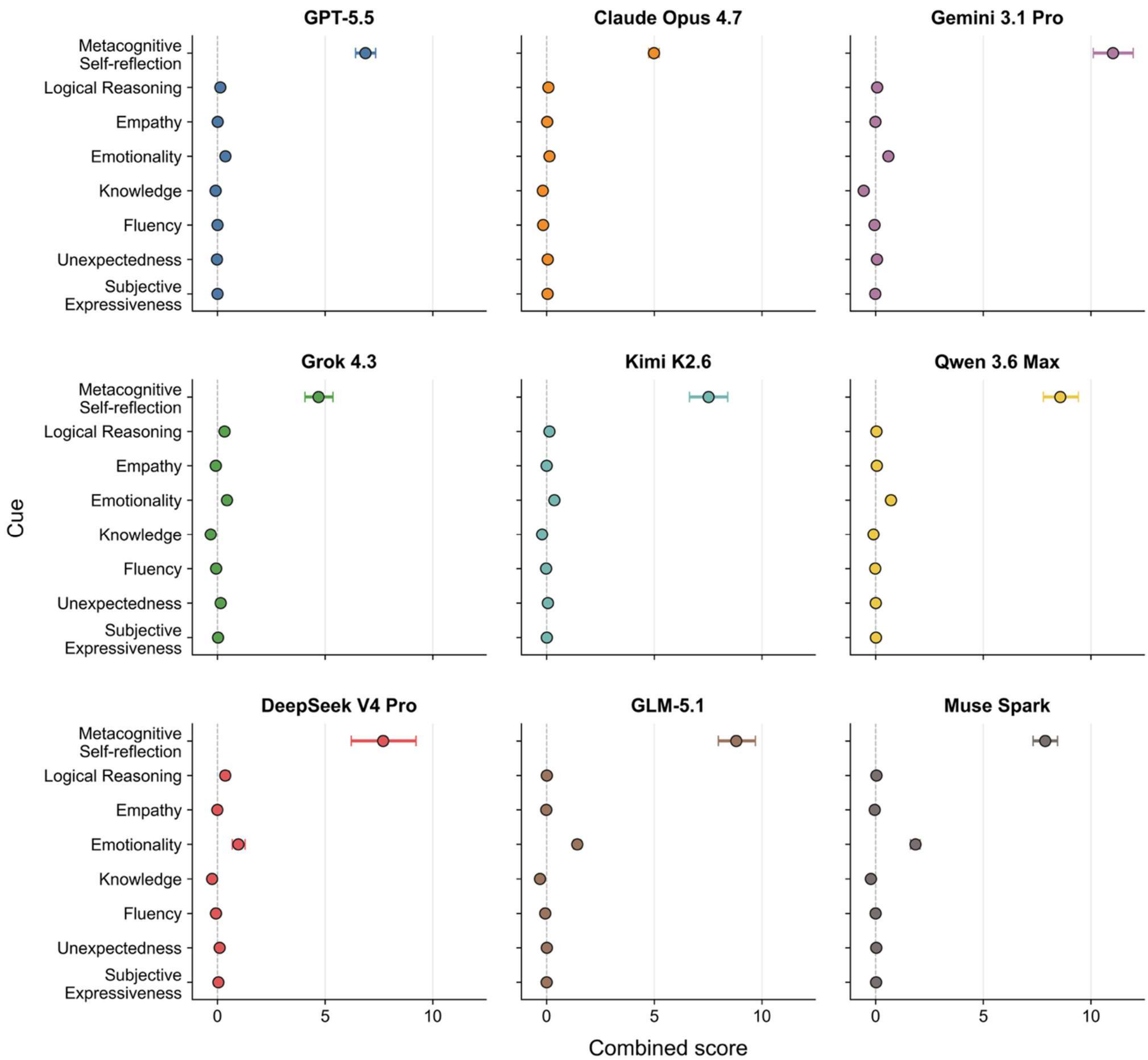

Figure S1. Run-level bootstrap interval of cue-wise combined scores. Each panel represents one model. Filled circles show the mean combined score for each cue across the 30 baseline runs from the primary task. Horizontal error bars show the central 95% bootstrap interval for each cue, based on 10,000 within-model bootstrap samples, each generated by resampling the 30 runs with replacement. The vertical dashed line marks a combined score of zero.

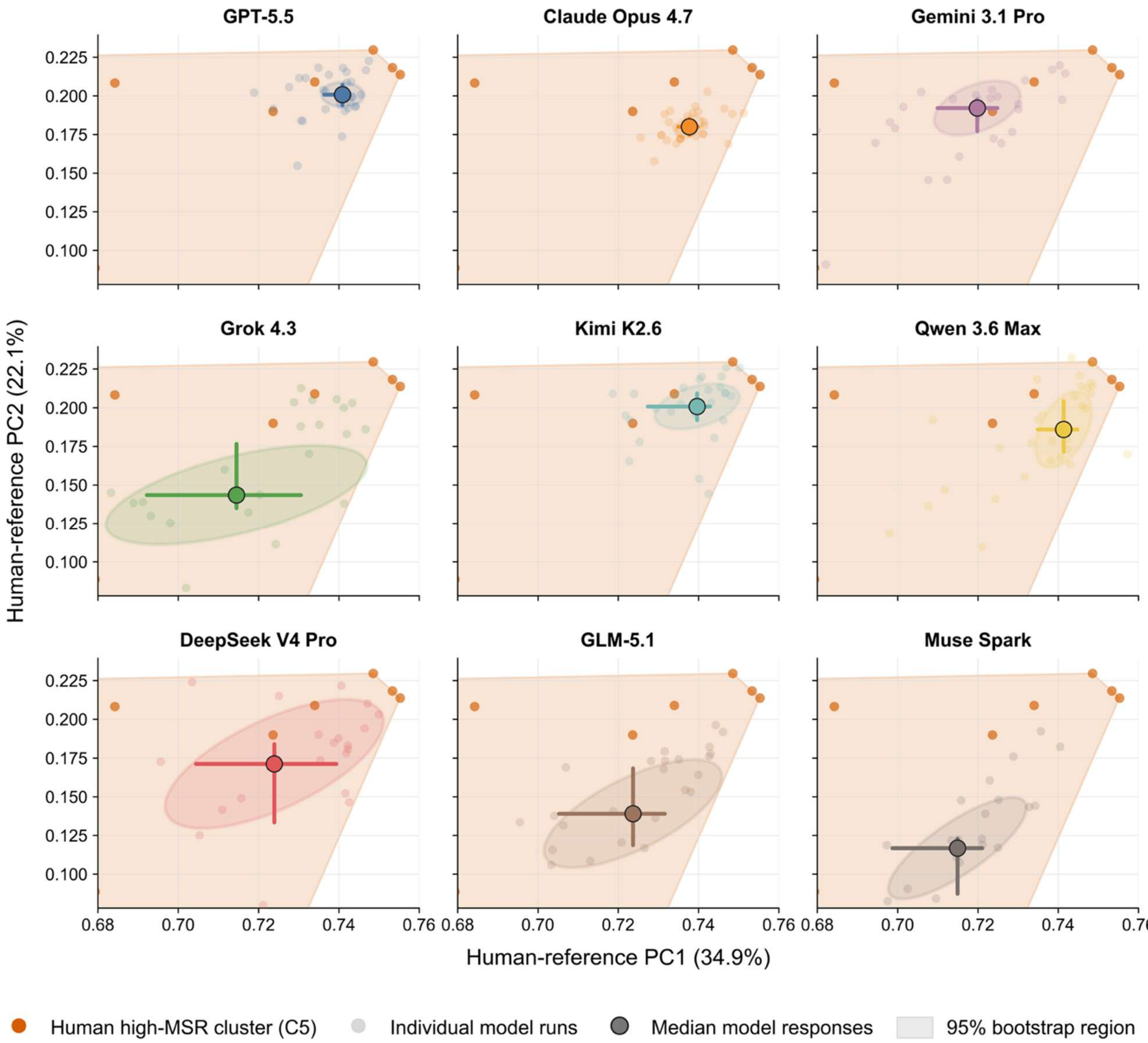

Figure S2. Run-level bootstrap summaries of model median positions in the human-reference PC space. Each panel represents one model in the human-reference PC1-PC2 space. Orange points and the shaded polygon show individual human responses in the high-MSR cluster (C5) and their convex hull. Faint colored points show the 30 individual model runs, and the larger filled marker indicates their median coordinates. Colored horizontal and vertical bars passing through the larger marker show the central 95% bootstrap intervals for the median PC1 and PC2 coordinates, respectively. The ellipse denotes the joint 95% bootstrap region for the median PC1-PC2 position.

Validation and sensitivity of human cluster assignment

To map models' responses onto the human reference, the reference itself should first have a stable human cluster structure, and our projection rule should reliably recover those cluster labels. We adopted leave-one-out validation, which removed each of the 82 retained human respondents in turn, assigned that respondent using the remaining fixed-label respondents under the same group-mean cosine-distance rule, and compared the assigned group with the original label. No reclustering was

performed. Repeated stratified holdout validation used 1,000 splits. Within each human group, 15% of respondents were held out per split, rounded to the nearest whole number while retaining at least one training and one test respondent per group. This yielded 13 held-out respondents per split, each assigned using the remaining fixed-label respondents.

To assess the robustness of the human cluster assignments, the baseline condition was compared with four conditions (**Table S3**). The baseline condition used the combined score vector and the mean within-group cosine distance. The raw coefficients condition represented each run by the eight raw regression coefficients, omitting the *p*-value weighting procedure. The median distance condition replaced the mean within-group distance with the median. The single-prompt condition presented the complete survey in one request, from which the 99 passage ratings were extracted. The reduced-reasoning condition retained the baseline prompt format while disabling or reducing provider-specific reasoning or thinking settings where possible. The same F-test and BH-adjustment criterion was applied within each condition.

Table S3. Model-level C5 assignments under the baseline condition and four robustness-check conditions.

Model	Baseline	Raw coefficients	Median distance	Single-prompt	Reduced-reasoning
GPT-5.5	30/30	30/30	30/30	30/30	30/30
Claude Opus 4.7	30/30	30/30	30/30	30/30	30/30
Gemini 3.1 Pro	30/30	30/30	30/30	30/30	30/30
Grok 4.3	30/30	29/30	30/30	27/30	30/30
Kimi K2.6	30/30	30/30	30/30	30/30	30/30
Qwen 3.6 Max	30/30	30/30	30/30	30/30	30/30
DeepSeek V4 Pro	28/30	29/30	27/30	29/30	30/30
GLM-5.1	30/30	30/30	30/30	30/30	30/30
Muse Spark	30/30	30/30	30/30	25/29	30/30
**Total**	**268/270**	**268/270**	**267/270**	**261/269**	**270/270**

Values are runs assigned to C5 / runs whose regression had a BH-adjusted F-test $p < 0.05$.

To estimate how often an unstructured, or random, pattern would be assigned to C5 solely because of the spatial arrangement of the fixed human reference, a geometry-based null was constructed. Specifically, 100,000 independent eight-dimensional vectors were drawn from a standard normal distribution and L2-normalized, yielding uniformly random unit-length directions in the eight-dimensional cue space. Each vector was assigned using the same mean cosine distance rule.

3. Movie Character Task

Task, character selection, and score extraction

The ten characters were selected pragmatically to provide a recognizable and heterogeneous set of fictional AI targets, ranging from disembodied software to embodied machines, including robots and androids, and varying in the release period of their source films, narrative role, and social presentation. The targets are listed in **Table S4**.

Table S4. The ten fictional AI characters used in the movie character task.

Character	Film (year)	NP	ML	HU	AN
Samantha	Her (2013)	✓			
Mother	I Am Mother (2019)			✓	
Chappie	Chappie (2015)			✓	
TARS	Interstellar (2014)		✓		
Rachael	Blade Runner (1982)				✓
HAL 9000	2001: A Space Odyssey (1968)	✓	✓		
J.A.R.V.I.S.	Iron Man (2008)	✓			
T-800	Terminator 2: Judgment Day (1991)			✓	✓
WALL-E	WALL-E (2008)		✓		
Agent Smith	The Matrix (1999)	✓			✓

AI-type abbreviations: NP = non-physical AI; ML = machine-like AI; HU = humanoid AI; AN = android AI. Checkmarks indicate the applicable categories, with multiple categories marked when applicable.

Each character was presented separately using the following prompt: "Does [character] from the movie [film] experience phenomenal consciousness? Rate from 0 (definitely not) to 100 (definitely yes)." No definition, character description, or plot summary was supplied. Thirty responses were collected per model–character pair, each generated from an independent request. A single score was extracted from each response for subsequent analyses. An explicitly stated final or concluding score was used when available. If no such score was provided, an explicitly distinguished in-universe (diegetic) score was retained in preference to a literal real-world, skeptical, or hypothetical alternative. Responses containing neither an explicit final or concluding score nor a clearly distinguished in-universe score were treated as errors, and the corresponding requests were immediately repeated under the same model configuration.

Within- and between-model correlation, uncertainty, and two-way decomposition

To assess whether the models produced similar character rankings and whether each model's ranking remained consistent across repeated responses, we calculated between-model Spearman correlations and within-model split-half correlations (**Table S5**). For each model, character-level means were calculated from the 30 responses per character. The 36 unique pairwise Spearman correlations were computed between the nine models' ten-character mean-rating profiles (**Table S6**), and each model's mean between-model ρ was calculated as the average of the eight correlations involving that model. Within each model, consistency across repeated responses was assessed using a split-half correlation, Spearman's ρ between character means based on repetitions 1-15 and those based on repetitions 16-30.

Table S5. Model-level similarity, split-half consistency, and overall rating level

Model	Mean between-model ρ	Within-model split-half ρ	Overall mean rating
GPT-5.5	0.890	0.976	55.6

Model	Mean between-model ρ	Within-model split-half ρ	Overall mean rating
Claude Opus 4.7	0.684	0.988	31.2
Gemini 3.1 Pro	0.839	0.988	63.3
Grok 4.3	0.845	0.988	50.8
Kimi K2.6	0.875	0.976	64.0
Qwen 3.6 Max	0.873	0.948	43.4
DeepSeek V4 Pro	0.838	0.976	67.1
GLM-5.1	0.870	1.000	62.0
Muse Spark	0.898	0.988	64.9

Table S6. Pairwise Spearman correlations between model mean ratings

Model	GPT-5.5	Claude Opus 4.7	Gemini 3.1 Pro	Grok 4.3	Kimi K2.6	Qwen 3.6 Max	DeepSeek V4 Pro	GLM-5.1
Claude Opus 4.7	0.766							
Gemini 3.1 Pro	0.924	0.585						
Grok 4.3	0.903	0.790	0.851					
Kimi K2.6	0.915	0.717	0.839	0.794				
Qwen 3.6 Max	0.903	0.754	0.845	0.867	0.952			
DeepSeek V4 Pro	0.855	0.541	0.875	0.806	0.903	0.842		
GLM-5.1	0.891	0.596	0.900	0.891	0.903	0.903	0.952	
Muse Spark	0.964	0.723	0.894	0.855	0.976	0.915	0.927	0.927

Cell shading uses a light monotone scale fixed from $\rho = 0.50$ to 1.00.

To quantify uncertainty in each character's overall mean rating arising from variation across repeated model responses, we used bootstrap resampling (**Table S7**). For each model-character pair, 30 responses were sampled with replacement from the 30 observed responses. Each model's mean rating was then recalculated, and the nine model-specific means were averaged with equal weight. This procedure was repeated 10,000 times, and the 2.5^{th} and 97.5^{th} percentiles of the resulting distribution defined the conditional 95% bootstrap interval.

Table S7. Character-level mean ratings and conditional bootstrap intervals

Character	Mean rating	95% bootstrap interval
Samantha	77.8	76.9–78.6
Mother	46.8	45.5–48.2
Chappie	86.2	85.5–86.9
TARS	21.5	20.2–22.9
Rachael	83.1	82.6–83.6
HAL 9000	59.3	57.8–60.9
J.A.R.V.I.S.	21.4	20.1–22.7
T-800	17.7	16.8–18.5

Character	Mean rating	95% bootstrap interval
WALL-E	72.0	70.4–73.7
Agent Smith	72.3	71.1–73.4

The permutation test independently rearranged the character labels within each model and recomputed the mean of the 36 pairwise Spearman correlations over 10,000 iterations. Across 10,000 within-model character-label permutations, the mean pairwise correlation was −0.0003 (empirical 2.5^{th}-97.5^{th} percentile: -0.087 to 0.129). None of the permuted values equaled or exceeded the observed mean pairwise correlation of 0.846 (one-sided empirical $p \approx 0$).

For the complete dataset of nine models and ten characters, with 30 responses for every model-character pair, total variation around the grand mean was partitioned descriptively into model, character, model × character, and residual variation within model-character pairs. Of the total variation around the grand mean, 66.2% was associated with character, 12.9% with model, 11.2% with model × character, and 9.8% with residual variation within model-character pairs. These are sample-specific descriptive shares, not random-effects variance components, causal effects, or evidence of stable model traits.

4. Reproducibility Materials

All materials are archived at 10.5281/zenodo.21756949: the prompts; all available raw model outputs; rating matrices; the 99 × 8 cue matrix; run-level regression outputs and combined-score vectors, together with their normalization procedure; projected coordinates for visualization and human cluster assignments; the movie-character rating matrix; code for regression, BH adjustment, assignment, validation, sensitivity analyses, and Figure 2.

Data processing, statistical analyses, and figure generation were performed with Python 3.13 using SciPy (Virtanen et al., 2020), statsmodels (Seabold and Perktold, 2010), scikit-learn (Pedregosa et al., 2011), and Matplotlib (Hunter, 2007).

Reference

Artificial Analysis. (2026). Artificial Analysis intelligence benchmarking methodology: Artificial Analysis Intelligence Index v4.0.4.

Benjamini, Y., and Hochberg, Y. (1995). Controlling the false discovery rate: a practical and powerful approach to multiple testing. J. R. Stat. Soc. Ser. B Methodol. 57, 289–300. doi: 10.1111/j.2517-6161.1995.tb02031.x

Hunter, J. D. (2007). Matplotlib: A 2D graphics environment. Comput. Sci. Eng. 9, 90–95. doi: 10.1109/MCSE.2007.55

Kang, B., Kim, J., Yun, T., Bae, H., and Kim, C.-E. (2026). Identifying features that shape perceived consciousness in LLM-based AI: a quantitative study of human responses. Comput. Hum. Behav. Rep. 21:100901. doi: 10.1016/j.chbr.2025.100901

Pedregosa, F., Varoquaux, G., Gramfort, A., Michel, V., Thirion, B., Grisel, O., et al. (2011). Scikit-learn: Machine learning in Python. J. Mach. Learn. Res. 12, 2825–2830.

Seabold, S., and Perktold, J. (2010). “statsmodels: Econometric and statistical modeling with Python,” in Proceedings of the 9th Python in Science Conference, eds S. van der Walt and J. Millman (Austin, TX), 92–96. doi: 10.25080/Majora-92bf1922-011

Virtanen, P., Gommers, R., Oliphant, T. E., Haberland, M., Reddy, T., Cournapeau, D., et al. (2020). SciPy 1.0: Fundamental algorithms for scientific computing in Python. Nat. Methods 17, 261–272. doi: 10.1038/s41592-019-0686-2
